# Somatostatin Receptor 2 Overexpression in Hepatocellular Carcinoma: Implications for Cancer Biology and Therapeutic Applications

**DOI:** 10.3390/curroncol32090512

**Published:** 2025-09-13

**Authors:** Servando Hernandez Vargas, Solmaz Aghaamiri, Jack T. Adams, Tyler M. Bateman, Belkacem Acidi, Sukhen C. Ghosh, Vahid Khalaj, Ahmed O. Kaseb, Hop S. Tran Cao, Majid Momeny, Ali Azhdarinia

**Affiliations:** 1 The Brown Foundation Institute of Molecular Medicine, McGovern Medical School, The University of Texas Health Science Center at Houston, Houston, TX 77030, USA; servando.hernandezvargas@uth.tmc.edu (S.H.V.); solmaz.aghaamiri@uth.tmc.edu (S.A.); sukhen.ghosh@uth.tmc.edu (S.C.G.); vahid.khalaj@uth.tmc.edu (V.K.); 2 Department of Surgical Oncology, Division of Surgery, The University of Texas MD Anderson Cancer Center, Houston, TX 77030, USAhstran@mdanderson.org (H.S.T.C.); 3 Department of Gastrointestinal Medical Oncology, The University of Texas MD Anderson Cancer Center, Houston, TX 77030, USA; akaseb@mdanderson.org

**Keywords:** hepatocellular carcinoma, SSTR2, tumorigenesis, overall survival

## Abstract

(1) Background: Somatostatin receptor 2 (SSTR2), a G protein-coupled receptor, is overexpressed in multiple malignancies, including hepatocellular carcinoma (HCC). While SSTR2 has traditionally been viewed as an inhibitory receptor involved in suppressing hormone secretion and cell proliferation, emerging evidence suggests a more complex role in cancer biology. However, the functional implications of SSTR2 expression in HCC remain poorly understood. This study aimed to systematically investigate the molecular landscape associated with SSTR2 expression in HCC and evaluate its potential as a therapeutic target. (2) Methods: SSTR2 expression patterns across 22 tumor types were assessed using TNMplot, and its expression in HCC was further validated through The Human Protein Atlas. Integrative analysis of transcriptomic profiles, protein expression data, and somatic copy number alterations was performed using data from The Cancer Genome Atlas (TCGA) to stratify HCC patients by SSTR2 expression levels. Gene Ontology (GO) enrichment analysis was conducted via SRplot to uncover biological processes and signaling pathways associated with SSTR2. Kaplan–Meier survival analyses were performed using GEO datasets to determine the prognostic significance of SSTR2 expression. (3) Results: SSTR2 is moderately expressed in the majority of HCC tumors. Elevated SSTR2 expression correlates with significantly poorer overall and disease-specific survival. High SSTR2 levels are associated with activation of oncogenic signaling cascades related to cell proliferation, epithelial-to-mesenchymal transition (EMT), angiogenesis, and metastasis. Additionally, SSTR2 expression is positively correlated with several receptor tyrosine kinases and oncogenes implicated in HCC progression. (4) Conclusions: Our findings suggest that SSTR2 is not merely a passive biomarker but may contribute to HCC pathogenesis through modulation of oncogenic pathways. These data support the rationale for further development of SSTR2-directed therapeutic strategies to inhibit tumor growth and invasion in HCC patients.

## Simple Summary

Liver cancer remains a major cause of death worldwide, and better ways to predict outcomes and guide treatment are urgently needed. This study focuses on somatostatin receptor 2, a protein on the surface of cells that senses hormone signals. Although this receptor is often thought to slow activity in normal tissues, its role in liver cancer has been unclear. We analyzed large publicly available collections of patient data to see how tumors with higher or lower amounts of somatostatin receptor 2 differ. We found that many liver cancers contain this receptor, and tumors with higher levels were linked to shorter survival. These tumors also showed stronger activity in genetic programs that drive cell growth, help cancer cells detach and spread to other organs, and build new blood vessels—features of more aggressive disease. Somatostatin receptor 2 was also associated with several well-known growth-promoting proteins in liver cancer. Taken together, our results suggest that somatostatin receptor 2 may contribute to liver cancer progression. This work highlights the receptor as a potential marker of risk and a candidate for therapies designed to block its signals, with the goal of slowing tumor growth and spread.

## 1. Introduction

Liver cancer, the fourth leading cause of cancer deaths and sixth in new cases globally, is expected to surpass one million cases by 2025 [1]. Hepatocellular carcinoma (HCC) is the most common primary liver malignancy, accounting for approximately 90% of liver cancer cases worldwide [2]. In the United States, HCC incidence has been steadily rising, with an estimated 42,600 new cases and 30,000 deaths in 2023, making it one of the most lethal cancers with a five-year survival rate of around 20% [3]. Major risk factors include chronic hepatitis B and C infections, alcohol-related liver disease, and non-alcoholic fatty liver disease associated with metabolic syndrome. 

Treatment options for HCC vary based on disease stage and include surgical resection, liver transplantation, locoregional therapies (radiofrequency ablation, transarterial chemoembolization), and systemic therapies, including multi-kinase inhibitors (sorafenib, lenvatinib) and immune checkpoint inhibitors [4]. However, HCC therapy faces significant challenges, including therapy resistance driven by tumor heterogeneity, hypoxia, and alterations in signaling pathways such as PI3K/AKT/mTOR and Wnt/β-catenin [5,6]. Additionally, a major clinical problem is the lack of reliable biomarkers for early detection and therapeutic response, coupled with our poor understanding of its molecular pathogenesis, which hinders the development of precision therapies [7].

Somatostatin receptor 2 (SSTR2) is one of the five known somatostatin receptor subtypes and is predominantly expressed in various tissues, including the central nervous system, endocrine glands, and gastrointestinal tract. SSTR2 plays a pivotal role in mediating the inhibitory effects of somatostatin on hormone secretion, cell proliferation, and neurotransmission. At the cellular level, SSTR2 is a G protein-coupled receptor (GPCR) that primarily couples with Gi proteins to inhibit adenylyl cyclase activity, leading to decreased cyclic AMP (cAMP) levels. This signaling cascade modulates ion channel activity and inhibits calcium influx, thereby reducing exocytosis of hormones and neurotransmitters. Molecularly, SSTR2 activation also influences downstream pathways such as the MAPK and PI3K/AKT signaling pathways, contributing to its role in regulation of cell cycle progression and apoptosis. Physiologically, SSTR2 regulates critical processes such as insulin and glucagon secretion in pancreatic islets, gastric acid secretion, and intestinal motility [8,9].

SSTR2 is overexpressed in several human cancers, including neuroendocrine tumors (NETs), small-cell lung carcinomas (SCLCs), and certain gliomas [8,9,10]. In HCC, nearly 40% of patients exhibit positive SSTR2 membrane staining, with intensities classified as strong (9.6%), moderate (21.2%), and weak (7.7%) [11]. Elevated expression of SSTR2 has been associated with favorable clinical outcomes in various human cancers. In rectal NETs, approximately two-thirds of patients exhibit SSTR2 expression, which correlates with smaller tumor size, lower tumor stage, and improved overall survival [12]. Similar correlation has been found in gliomas, where high SSTR2 levels are linked to lower WHO grades and better prognoses [10]. Conversely, high SSTR2 expression has been associated with poor prognosis in nasopharyngeal carcinoma [13]. Similarly, in SCLC, SSTR2 signaling has been linked to tumor progression [9], suggesting that SSTR2 may exhibit both oncogenic and tumor suppressor functions depending on the cancer type.

Although SSTR2 is significantly expressed in HCC, its relationship with the clinicopathological features of this cancer remains poorly characterized. Furthermore, the functional roles of SSTR2 in the molecular pathology of HCC and the signaling pathways it regulates are not yet fully understood. This study aims to provide preliminary evidence for the clinical relevance of SSTR2 in HCC and to investigate its potential as a therapeutic target in this malignancy.

## 2. Materials and Methods

### 2.1. Study Design and Data Sources

We conducted a retrospective integrative analysis using publicly available, de-identified datasets to evaluate associations between SSTR2 expression and clinical, transcriptomic, proteomic, and genomic features in HCC. Data sources were as follows: TNMplot (pan-cancer differential expression), The Human Protein Atlas (HPA) (IHC for SSTR2 in HCC), TCGA-LIHC via cBioPortal (RNA-seq counts, RPPA protein profiles, and arm-level copy-number alterations), SRplot (GO enrichment), and KMplot (pooled GEO survival datasets). No new experiments on humans or animals were performed.

- TNMplot (https://tnmplot.com)—accessed 20 August 2025
- HPA (https://www.proteinatlas.org)—accessed 20 August 2025
- TCGA-LIHC via cBioPortal (https://www.cbioportal.org)—accessed 21 August 2025 [14]
- SRplot (https://www.bioinformatics.com.cn/en)—accessed 22 August 2025 [15]
- KMplot (https://kmplot.com)—accessed 21 August 2025 [16]

### 2.2. Pan-Cancer and Protein Expression Assessment

**Figure 1:**
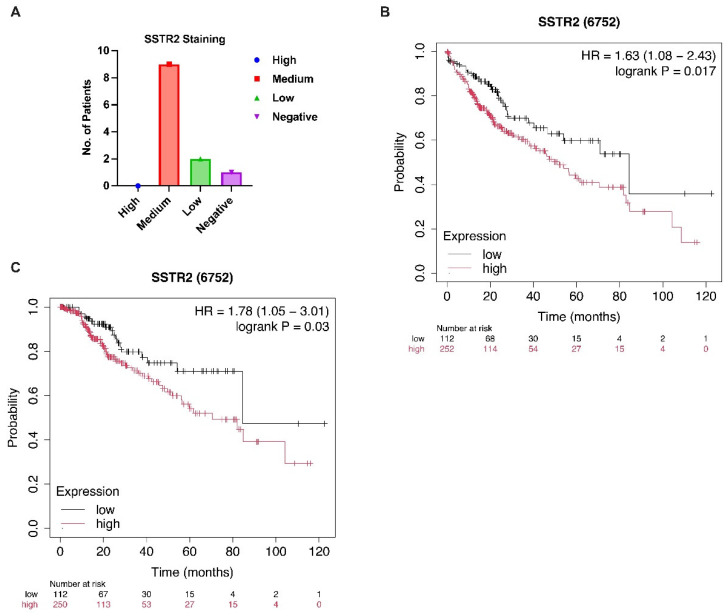
SSTR2 expression and its prognostic significance in HCC. (**A**) Representative IHC staining images showing SSTR2 protein expression in tumor tissues from 12 HCC patients. Staining intensity was predominantly moderate in the majority of cases, with 9 out of 12 patients exhibiting medium staining, 2 showing weak staining, and 1 with no detectable staining. Data were obtained from the Human Protein Atlas (HPA; https://www.proteinatlas.org) using antibody HPA007264. (**B**,**C**) Kaplan–Meier survival curves illustrating the association between SSTR2 mRNA expression and clinical outcomes in HCC patients. Patients with elevated SSTR2 expression exhibited significantly poorer overall survival (**B**) and disease-specific survival (**C**) compared to those with lower expression levels. Survival analysis was performed using the Kaplan–Meier Plotter (https://kmplot.com) based on publicly available transcriptomic datasets.

TNMplot was queried to compare *SSTR2* mRNA between tumors and matched/adjacent normal tissues across 22 cancers. For protein, HPA entries using antibody HPA007264 were reviewed for HCC; we recorded pathologist-annotated staining intensity (negative/weak/moderate/strong), percent positive cells, and subcellular localization for each available case. Representative images are shown in Figure 1A, and tallies are reported in Results.

### 2.3. TCGA-LIHC Cohort, Preprocessing, and Group Definition

Gene-level RNA-seq counts and clinical annotations for TCGA-LIHC were downloaded via cBioPortal. Samples with missing clinical metadata, duplicated barcodes, or low library complexity (library size below the 1st percentile) were excluded a priori. Counts were normalized to TPM and transformed as log2(TPM + 1) for visualization and correlation; for differential expression (DE), we used raw counts with model-based normalization (below).

Patients were stratified by *SSTR2* expression into quartiles. To increase contrast and avoid arbitrary cutoffs, we compared the upper quartile (Q4, “SSTR2^high^”) vs. lower quartile (Q1, “SSTR2^low^”), excluding the middle 50% from DE and pathway analyses. These same groups were used for RPPA and copy-number comparisons. Sample sizes for each stratum are reported in Results.

### 2.4. Differential Expression and Correlation Analyses

DE was computed using DESeq2 (R package DESeq2 v1.42.0) with default size-factor/dispersion estimation on raw counts, followed by Benjamini–Hochberg correction. Unless otherwise specified, significance thresholds were |log2 fold-change| ≥ 1 and FDR < 0.05. Gene–gene associations (e.g., with FGFR2, PDGFRA, and PDGFRB) were assessed on log2(TPM+1) values using Pearson’s r with BH adjustment across families of tests.

### 2.5. Functional Enrichment and GSEA

Upregulated genes in SSTR2^high^ tumors were analyzed with SRplot for Gene Ontology (GO) Biological Process enrichment (hypergeometric test with BH FDR). Where indicated, we performed Gene Set Enrichment Analysis (GSEA) using fgsea v1.28.0 against MSigDB Hallmark and curated collections retrieved via msigdbr v7.5.1. We report normalized enrichment scores (NESs) and FDR q-values, with pathways considered enriched at FDR < 0.05.

### 2.6. Proteomics (RPPA)

Protein abundance data (Pan-Cancer Atlas RPPA) for TCGA-LIHC were retrieved via cBioPortal. We compared SSTR2^high^ vs. SSTR2^low^ using two-sided Wilcoxon rank-sum tests with BH FDR correction. Proteins highlighted in the text (e.g., PAI-1, TIGAR, SYK, FN1, CCNB1, MAPK1, FASN, SRC) met FDR < 0.05 and showed coherent directionality with corresponding transcriptomic programs.

### 2.7. Copy-Number Alterations (CNA)

Arm-level GISTIC2.0 calls (−1 loss, +1 gain; −2 deep loss, +2 high-level gain) were obtained for TCGA-LIHC. We compared event frequencies between SSTR2^high^ and SSTR2^low^ cohorts using Fisher’s exact test (two-sided), reporting odds ratios and BH-adjusted q-values. Pre-specified attention was given to 1p and 16q given the presence of *CDH1* and *RUNX3* loci, respectively.

### 2.8. Survival Analyses

Overall survival (OS), disease-specific survival (DSS), progression-free survival (PFS), and relapse-free survival (RFS) were evaluated via KMplot using the “auto select best cutoff” disabled; we dichotomized by quartiles as above (Q4 vs. Q1). Curves were generated by the Kaplan–Meier method with log-rank tests. Where available, we computed univariable Cox proportional hazards models (R survival v3.5-7) to obtain HRs and 95% CIs. Given data aggregation constraints in KMplot, multivariable adjustment was not performed; we acknowledge this as a limitation and recommend validation in independent, covariate-adjusted cohorts.

### 2.9. Statistics, Software, and Reproducibility

All tests were two-sided. Multiple testing was controlled using Benjamini–Hochberg FDR unless specified. Analyses were performed in R v4.3.2 with the following core packages: DESeq2 v1.42.0, edgeR v3.42.4 (where exploratory dispersion checks were needed), fgsea v1.28.0, msigdbr v7.5.1, survival v3.5-7, survminer v0.4.9, and ggplot2 v3.5.1. Exact code and session info (package versions and seeds) are available upon request and will be deposited in a public repository upon acceptance.

### 2.10. Ethical Considerations

All analyses used de-identified, publicly available data and did not constitute human subjects research; IRB review was not required. Analyses complied with terms of use for each resource.

## 3. Results

Using the TNMplot web-based analytical tool, we evaluated the mRNA expression levels of *SSTR2* across 22 different human cancer types by comparing matched tumor and adjacent normal tissue samples. Our analysis demonstrated that SSTR2 transcript levels were significantly elevated in tumor tissues compared to their normal counterparts in several malignancies, including liver cancer (Supplementary Figure S1). This suggests that SSTR2 may be upregulated during malignant transformation and could play a context-specific role in tumor biology.

To further confirm the relevance of SSTR2 expression at the protein level in HCC, we examined immunohistochemistry (IHC) data from The Human Protein Atlas (HPA). IHC staining was performed using the anti-SSTR2 antibody HPA007264 (Sigma-Aldrich) on liver cancer tissues from 12 HCC patients. Among these cases, nine patients (75%) showed medium staining intensity, two patients (16.7%) exhibited weak staining, and one patient (8.3%) had no detectable SSTR2 protein expression (Figure 1A). Staining was primarily localized to the plasma membrane and cytoplasm, consistent with the known subcellular localization of SSTR2 as a G-protein-coupled receptor.

Given the observed elevated expression of *SSTR2* in liver cancer tissues compared to adjacent normal tissues (Supplementary Figure S1), we hypothesized that SSTR2 may play a functional role in HCC tumorigenesis. To explore the clinical significance of SSTR2 expression in HCC, we employed the Kaplan–Meier Plotter tool to assess the relationship between *SSTR2* mRNA levels and patient survival outcomes. The analysis revealed that higher *SSTR2* expression was significantly associated with poorer overall survival (OS) and disease-specific survival (DSS) in HCC patients (Figure 1B,C), suggesting that SSTR2 overexpression may have prognostic implications.

To further validate these observations, we analyzed RNA sequencing data from the TCGA-LIHC dataset [14], stratifying patients based on normalized *SSTR2* mRNA expression into two groups: SSTR2^high^ (log fold change [LogFC] > 1, false discovery rate [FDR] < 0.05; *n* = 60) and SSTR2^low^ (LogFC < –1, FDR < 0.05; *n* = 59). Kaplan–Meier survival analysis of these groups confirmed that patients in the SSTR2^high^ group exhibited significantly worse overall survival (Log-rank test *p* = 0.0393), reinforcing the association between elevated SSTR2 expression and poor clinical outcome (Supplementary Figure S2).

**Figure 2:**
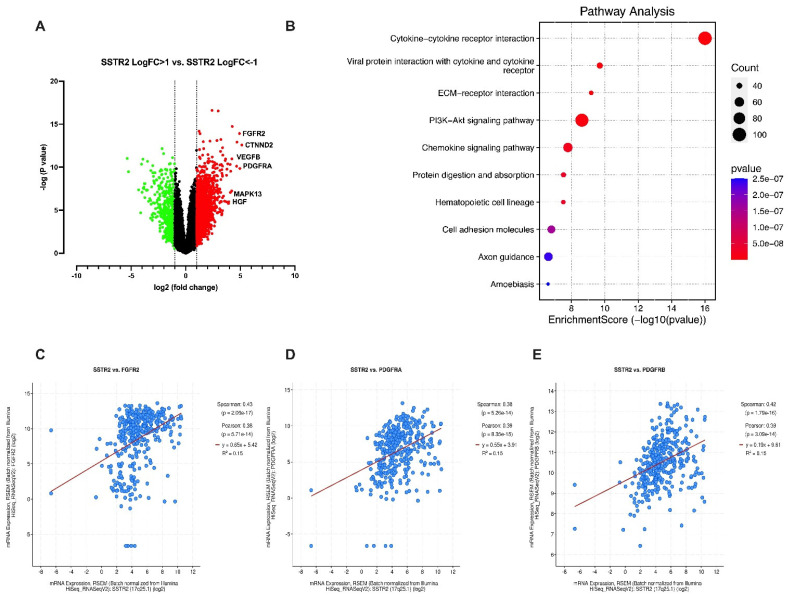
Transcriptomic profiling of SSTR2^high^ versus SSTR2^low^ HCC tumors reveals distinct oncogenic signatures. (**A**) Patients from the TCGA-LIHC dataset [14] were stratified into SSTR2^high^ (log fold change [LogFC] > 1, false discovery rate [FDR] < 0.05, *n* = 60) and SSTR2^low^ (LogFC < –1, FDR < 0.05, *n* = 59) groups based on normalized *SSTR2* mRNA expression levels. Differential gene expression analysis revealed significant alterations in the expression of multiple oncogenes associated with HCC progression and poor prognosis. (**B**) GSEA of upregulated genes in the SSTR2^high^ group identified enrichment of several tumor-promoting pathways, including chemokine signaling, PI3K/AKT signaling, cell adhesion, and tumor–stroma interactions, indicating a more aggressive molecular phenotype. (**C**–**E**) Pearson correlation analyses demonstrated a significant positive correlation between SSTR2 expression and mRNA levels of key receptor tyrosine kinases, including FGFR2 (**C**), PDGFRA (**D**), and PDGFRB (**E**) in HCC patients from the TCGA dataset. These findings suggest that SSTR2 overexpression is associated with the activation of RTK-driven oncogenic signaling networks.

Next, we performed a differential gene expression analysis to investigate the transcriptomic alterations associated with SSTR2 overexpression. Compared to the SSTR2^low^ group, the SSTR2^high^ group exhibited significantly higher expression of approximately 1,800 genes (LogFC > 1, *p* < 0.05) and lower expression of around 500 genes (LogFC < –1, *p* < 0.05) (Figure 2A). These differentially expressed genes reflect the altered molecular landscape in SSTR2^high^ tumors.

Gene Set Enrichment Analysis (GSEA) of the upregulated genes in the SSTR2^high^ group identified enrichment in several oncogenic and tumor-promoting pathways, including chemokine signaling, PI3K/AKT pathway activation, cell adhesion, extracellular matrix remodeling, and tumor–stroma interactions (Figure 2B). These pathways are known to contribute to tumor proliferation, immune evasion, angiogenesis, and metastatic potential in HCC [17,18], suggesting that SSTR2 may promote a more aggressive tumor phenotype through modulation of these signaling networks. Collectively, these findings support the notion that SSTR2 expression is not only a prognostic biomarker in HCC but also potentially contributes to disease progression by regulating key oncogenic pathways.

Among the genes found to be significantly upregulated in the SSTR2^high^ group, several are known to play critical roles in tumor progression, angiogenesis, and metastasis. Specifically, delta-catenin (CTNND2), vascular endothelial growth factor B (VEGFB), platelet-derived growth factor receptor alpha (PDGFRA), mitogen-activated protein kinase 13 (MAPK13), and hepatocyte growth factor (HGF) all exhibited markedly elevated expression levels in SSTR2^high^ tumors (Figure 2A). These molecules are well-characterized drivers of oncogenic signaling in HCC and have been individually implicated in promoting tumor cell survival, proliferation, angiogenesis, and metastasis [19].

To further explore potential mechanistic relationships, we assessed the correlation between SSTR2 mRNA expression and key receptor tyrosine kinases (RTKs) in the TCGA-LIHC dataset [14]. Our analysis revealed a significant positive correlation between SSTR2 expression and PDGFRA, PDGFRB (platelet-derived growth factor receptor beta), and FGFR2 (fibroblast growth factor receptor 2) (Figure 2C–E). These RTKs are known to activate multiple downstream signaling cascades, including PI3K/AKT, RAS/MAPK, and JAK/STAT, all of which are frequently dysregulated in HCC and contribute to resistance to conventional therapies [20]. 

Importantly, elevated levels of VEGFB, PDGFRA, MAPK13, and HGF have been previously linked to poor prognosis and aggressive tumor behavior in HCC [21]. These factors enhance tumor vascularization, promote immune evasion, and support metastatic dissemination. In parallel, delta-catenin (CTNND2), a member of the p120-catenin family, plays a critical role in modulating cell–cell adhesion and has emerged as a key regulator of epithelial–mesenchymal transition (EMT)—a cellular program that endows epithelial cancer cells with mesenchymal traits, thereby enhancing their migratory and invasive capabilities [22,23]. 

**Figure 3:**
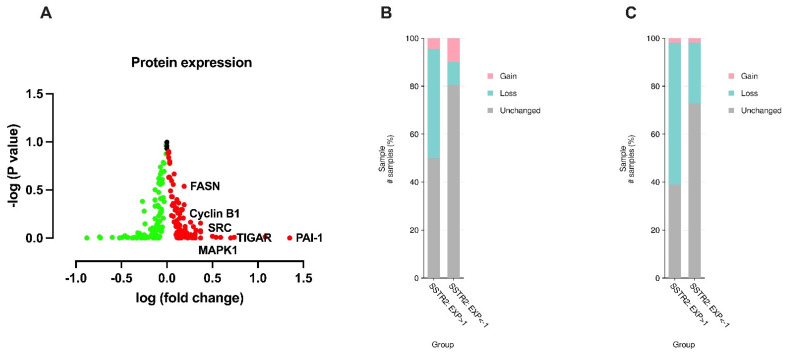
Proteogenomic characterization of SSTR2^high^ HCC reveals enhanced oncogenic signaling and chromosomal instability. (**A**) Comparative protein expression analysis using RPPA data from TCGA-LIHC [14] patients stratified by *SSTR2* mRNA expression revealed significantly elevated levels of multiple oncogenic proteins in the SSTR2^high^ group, including plasminogen activator inhibitor-1 (PAI-1), TP53-induced glycolysis and apoptosis regulator (TIGAR), spleen tyrosine kinase (SYK), fibronectin 1 (FN1), cyclin B1, MAPK1 (ERK2), fatty acid synthase (FASN), and SRC. These proteins are known to promote cell survival, EMT, tumor proliferation, and invasion, contributing to a more aggressive HCC phenotype. (**B**,**C**) Arm-level copy number alteration (CNA) analysis showed a significantly higher frequency of chromosomal losses at 1p and 16q in the SSTR2^high^ group compared to SSTR2^low^ patients. These genomic deletions encompass key tumor suppressor genes, including CDH1 (1p22.1), which encodes E-cadherin, and RUNX3 (16q24.1). Both genes are implicated in suppressing EMT, maintaining epithelial integrity, and inhibiting tumor invasion and metastasis. The enrichment of these losses in SSTR2^high^ tumors supports their association with genomic instability and a more invasive disease phenotype.

To gain further insights into the biological differences associated with SSTR2 expression levels in HCC, we compared the protein expression profiles of the SSTR2^high^ and SSTR2^low^ groups using reverse-phase protein array (RPPA) data from the TCGA-LIHC cohort [14]. This analysis revealed a set of oncogenic proteins that were significantly upregulated in the SSTR2^high^ group, including plasminogen activator inhibitor-1 (PAI-1), TP53-induced glycolysis and apoptosis regulator (TIGAR), spleen tyrosine kinase (SYK), fibronectin 1 (FN1), cyclin B1 (CCNB1), MAPK1 (ERK2), fatty acid synthase (FASN), and SRC proto-oncogene (Figure 3A). These proteins are known to be critical regulators of tumor growth, metabolic reprogramming, cell cycle progression, extracellular matrix remodeling, and metastasis in HCC and other solid tumors.

PAI-1, a serine protease inhibitor, plays a central role in extracellular matrix degradation and tissue remodeling, thereby facilitating tumor invasion and metastatic spread. Its overexpression has been correlated with increased invasiveness and poor prognosis in HCC [24]. Similarly, TIGAR, a downstream effector of p53, regulates glycolysis and antioxidant defense; its upregulation promotes cell survival, while *TIGAR* knockdown has been shown to induce apoptosis and autophagy in HCC cells, suggesting its role in metabolic adaptation and therapy resistance [25].

SYK, a non-receptor tyrosine kinase, has emerged as a key player in EMT, tumor metastasis, and vascular invasion [26], all of which are hallmark features of aggressive HCC. FN1, a major component of the extracellular matrix, has also been implicated in the induction of EMT in liver cancer, contributing to enhanced motility and invasive behavior of tumor cells [27]. 

Furthermore, cyclin B1, MAPK1, and SRC are central mediators of cell cycle progression, MAPK signaling, and oncogenic kinase activity, respectively. Their overexpression is consistently associated with enhanced tumor proliferation, resistance to apoptosis, and poor clinical outcomes in HCC [28,29,30]. FASN, a key enzyme involved in de novo lipogenesis, supports the high metabolic demands of cancer cells and has been shown to correlate with tumor aggressiveness and reduced survival in liver cancer patients [31]. Together, these findings underscore that high SSTR2 expression in HCC is associated with a proteomic landscape enriched for oncogenic signaling and markers of tumor progression, further supporting the hypothesis that SSTR2^high^ tumors represent a biologically aggressive and clinically unfavorable subtype of HCC.

To further explore the genomic alterations associated with SSTR2 expression in HCC, we performed a comparative analysis of arm-level copy number alterations (CNAs) between the SSTR2^high^ and SSTR2^low^ patient groups using TCGA-LIHC data [14]. This analysis revealed that SSTR2^high^ tumors exhibited significantly higher frequencies of chromosomal losses in 1p and 16q arms relative to the SSTR2^low^ group (Figure 3B,C), indicating that specific genomic instability may underlie the aggressive behavior of SSTR2-overexpressing tumors.

Among the genes located in the 1p22.1 region, CDH1, which encodes E-cadherin, was of particular interest. E-cadherin is a key component of adherens junctions and plays a fundamental role in maintaining epithelial integrity, polarity, and suppressing metastasis. Loss of CDH1 function, either through mutation, epigenetic silencing, or chromosomal deletion, has been extensively linked to EMT and enhanced invasive potential in HCC and other epithelial malignancies [32]. Therefore, the observed 1p arm-level deletion in SSTR2^high^ tumors suggests a molecular mechanism through which these tumors may acquire mesenchymal features and increased metastatic capacity.

Similarly, the 16q24.1 region, frequently lost in SSTR2^high^ patients, harbors RUNX3, a well-established tumor suppressor transcription factor. RUNX3 has been shown to promote CDH1 expression and inhibit EMT and cell migration, thereby restraining tumor invasion and progression [33]. Deletion or downregulation of RUNX3 in HCC is associated with increased tumor aggressiveness, vascular invasion, and worse prognosis [34]. Thus, the loss of 16q in SSTR2^high^ tumors likely exacerbates the loss of epithelial characteristics and reinforces a transcriptional program favoring invasion and dissemination.

## 4. Discussion

Our study suggests that high SSTR2 expression in HCC could significantly impact its clinical management, serving as both a prognostic biomarker for patient outcomes and a therapeutic target for treatment purposes. Our novel observations suggest a potential link between SSTR2 expression, receptor tyrosine kinase expression, tumor metastasis, clinical outcomes, and therapeutic sensitivity. 

Our findings about SSTR2 expression in HCC align with previous IHC studies that have reported detectable SSTR2 expression in a subset of HCC tumors. For instance, prior work indicated that approximately 40% of HCC cases express SSTR2, with 9.6% showing strong membrane staining, 21.2% moderate, and 7.7% weak staining [11]. The variability in staining intensity observed across different patient samples suggests heterogeneous SSTR2 expression within HCC, which may have implications for stratifying patients for targeted therapies and for understanding tumor biology in subgroups with high SSTR2 expression. Collectively, these data provide converging evidence from transcriptomic and proteomic levels that SSTR2 is frequently, though variably, expressed in HCC, supporting its further investigation as a clinically relevant biomarker and potential therapeutic target.

The VEGFR, PDGFR, and RAF/MEK/ERK signal transduction pathways are integral to the pathogenesis and progression of HCC. VEGFRs are key regulators of angiogenesis, which facilitates tumor growth and metastasis by promoting blood vessel formation in HCC [35]. PDGFRs play crucial roles in tumor–stroma interactions, contributing to HCC progression through enhanced stromal support, angiogenesis, and fibrogenesis [36,37]. Additionally, the RAF/MEK/ERK pathway, a central mediator of the MAPK signaling cascade, is often dysregulated in HCC, driving cellular proliferation, survival, and resistance to apoptosis [30]. Dysregulation of these pathways promotes HCC tumorigenesis and their blockade, either individually or in combination, and holds promise as a therapeutic strategy to inhibit tumor growth and malignant progression in HCC [17]. Together, these findings suggest that high SSTR2 expression is associated with a molecular profile enriched for pro-tumorigenic and pro-metastatic signaling pathways, reinforcing its potential role as a functional driver of HCC progression. These correlations also raise the possibility that SSTR2 may interact with or modulate RTK-mediated signaling networks, thereby contributing to the aggressive phenotype observed in SSTR2^high^ tumors.

Sorafenib, an FDA-approved multi-kinase inhibitor for HCC, exerts its anti-tumor effects by inhibiting cell proliferation and angiogenesis through targeting the VEGFR, PDGFR, and RAF/MEK/ERK pathways [38]. The findings of this study reveal a positive correlation between high SSTR2 expression and the upregulation of these key oncogenic pathways in HCC. However, whether SSTR2 directly regulates these pathways or whether they collectively contribute to an oncogenic network in HCC remains an open and intriguing question. Addressing this question could unveil new opportunities for prognosis and therapeutic intervention. SSTR2 may play a role in the molecular pathology of HCC by regulating these oncogenic pathways, making it a promising novel therapeutic target for the treatment of HCC. Moreover, targeting SSTR2 with specific modulators may disrupt the VEGFR, PDGFR, and RAF/MEK/ERK pathways, offering a synergistic approach with sorafenib to inhibit tumor growth and angiogenesis more effectively.

Despite its broad spectrum of molecular targets, the clinical efficacy of sorafenib in HCC remains limited. Clinical trials report that only approximately 30% of patients derive substantial benefit, with a median overall survival improvement of just 2–3 months. Moreover, resistance to sorafenib frequently develops within six months of treatment initiation, underscoring the roles of both intrinsic and acquired resistance mechanisms [39,40]. These limitations have spurred significant research efforts to enhance the therapeutic effectiveness of sorafenib. Combination therapies have emerged as promising strategies to overcome resistance and achieve synergistic anti-tumor effects [24]. While the role of SSTR2 in mediating resistance to sorafenib in HCC remains to be elucidated, SSTR2-targeted theranostic applications have significant potential to synergize with sorafenib and improve its efficacy in sorafenib-resistant SSTR2-positive cases. 

Mechanistically, pairing SSTR2-directed theranostic radioligands with sorafenib is plausible via three testable axes: ^177^Lu-DOTATATE-driven DNA damage/ROS layered onto sorafenib’s mitochondrial/redox stress; multi-kinase blockade (RAF/VEGFR/PDGFR) reducing pro-survival signaling while RAD51 targeting limits repair of PRRT-induced breaks; and anti-angiogenic vascular-normalization windows that can boost oxygenation and radiation effectiveness with proper timing. Clinically, sorafenib has combined feasibly with radiotherapy; SSTR2-positive HCC offers a route for targeted radioligand delivery, warranting formal synergy testing. [41,42,43]. Supporting this hypothesis, our recent findings demonstrate that ^177^Lu-DOTATATE enhances the anti-tumor activity of sorafenib in an HCC cell line [44]. 

These co-occurring chromosomal losses in SSTR2-overexpressing tumors are consistent with a model in which SSTR2 upregulation is associated with a distinct genomic instability pattern that promotes metastatic transformation. The enrichment of deletions in tumor suppressor loci involved in cell adhesion and EMT regulation further supports the hypothesis that SSTR2 expression is not merely a biomarker but may reflect or contribute to a genetically and phenotypically aggressive subtype of HCC. Together, these results provide a molecular explanation for the poor clinical outcomes observed in SSTR2^high^ HCC patients, suggesting that SSTR2 expression is intricately linked with a loss of cell adhesion, EMT activation, and tumor invasiveness, and may serve as a surrogate indicator of aggressive genomic alterations in liver cancer.

Another noteworthy finding of this study is the elevated expression of key proteins involved in extracellular matrix degradation, motility, and invasion in HCC patients with higher SSTR2 expression, including PAI-1, TIGAR, SYK, fibronectin, MAPK1, FASN, and SRC. Consistently, we observed a higher frequency of deletions in CDH1, which encodes E-cadherin, a critical suppressor of tumor metastasis [32], and RUNX3, which promotes E-cadherin expression and inhibits EMT [33], in these patients. The loss of E-cadherin and activation of pro-metastatic pathways, including SRC and MAPK, further accelerates invasion and metastasis in HCC [45,46,47]. 

Metastasis, a critical driver of poor prognosis in HCC, involves complex processes such as EMT, ECM degradation, angiogenesis, and immune evasion. These mechanisms facilitate tumor dissemination to distant organs, such as the lungs and lymph nodes, and correlate with aggressive tumor behavior, therapy resistance, and reduced survival [48]. These co-occurring chromosomal losses in SSTR2-overexpressing tumors are consistent with a model in which SSTR2 upregulation is associated with a distinct genomic instability pattern that promotes metastatic transformation. The enrichment of deletions in tumor suppressor loci involved in cell adhesion and EMT regulation further supports the hypothesis that SSTR2 expression is not merely a biomarker but may reflect or contribute to a genetically and phenotypically aggressive subtype of HCC. Moreover, these results provide a molecular explanation for the poor clinical outcomes observed in SSTR2^high^ HCC patients, suggesting that SSTR2 expression is intricately linked with loss of cell adhesion, EMT activation, and tumor invasiveness, and may serve as a surrogate indicator of aggressive genomic alterations in liver cancer.

Collectively, these insights highlight the importance of further investigations to elucidate the role of SSTR2 in cancer metastasis in HCC and to explore its potential as a therapeutic target to inhibit tumor invasion in this highly fatal malignancy. These preliminary results warrant further validation through advanced animal models and early-phase clinical trials to explore their translational potential.

### Limitations and Future Directions

This integrative analysis of public, de-identified datasets is correlative and does not establish causality. To validate the associations, causality should be tested by SSTR2 loss- and gain-of-function in HCC models with readouts of proliferation, apoptosis, migration/invasion, and MAPK/ERK and PI3K/AKT signaling, and by probing ligand dependence (somatostatin analogs/antagonists) with internalization/β-arrestin assays. Therapeutic relevance should be examined by pairing SSTR2 modulation or SSTR2-directed theranostics with multikinase inhibitors (e.g., sorafenib), using formal drug–interaction matrices alongside rescue experiments and RNA-seq/phosphoproteomics to map downstream effectors; confirmatory xenograft/orthotopic studies may be warranted. Finally, prognostic value should be reassessed in independent cohorts using multivariable models that adjust for clinical and genomic covariates.

## 5. Conclusions

Our study provides compelling evidence that SSTR2 is not merely an overexpressed marker in HCC but may participate in a broader oncogenic signaling network that contributes to tumor progression, invasion, and poor clinical outcomes. Elevated SSTR2 expression in HCC is associated with transcriptomic and proteomic signatures linked to proliferation, EMT, angiogenesis, receptor tyrosine kinase signaling, and chromosomal instability. In particular, SSTR2^high^ tumors demonstrate upregulation of genes and proteins that promote tumor aggressiveness and show enrichment in genomic alterations such as losses in 1p and 16q, which further drive metastatic potential.

Clinically, these findings have important implications for diagnosis, prognosis, and therapeutic intervention. HCC patients with elevated SSTR2 expression may be ideal candidates for ^68^Ga-DOTATATE PET/CT imaging, a molecular imaging modality that can provide superior tumor detection, evaluate treatment response, and guide patient stratification for targeted therapies [49]. The use of ^68^Ga-DOTATATE PET/CT, which is already approved for NETs [50], could be explored in selected SSTR2-positive HCC, with prospective evaluation of diagnostic performance and clinical utility. Therapeutically, these patients may also benefit from peptide receptor radionuclide therapy (PRRT) using ^177^Lu-DOTATATE, which delivers targeted beta-emitting radiation to SSTR2-expressing tumor cells [51]. Although ^177^Lu-DOTATATE has been approved for advanced NETs [52], its potential utility in HCC remains underexplored. Our data strongly support further preclinical and clinical evaluation of SSTR2-directed PRRT in liver cancer, particularly for tumors that demonstrate high SSTR2 expression and co-expression of actionable oncogenic targets.

In addition to monotherapies, the integration of SSTR2-targeted radiopharmaceuticals with immunotherapies, multi-kinase inhibitors, or chemotherapeutic agents warrants investigation, particularly in the context of overcoming resistance mechanisms and enhancing treatment durability. The variability in SSTR2 staining intensity among HCC patients may reflect underlying intratumoral heterogeneity, which has important clinical implications. ^68^Ga-DOTATATE enables noninvasive assessment of SSTR2 expression and patients with strong expression may be better candidates for PRRT. Conversely, those with weak or heterogeneous staining may require combinatorial approaches, such as pairing PRRT with immune checkpoint inhibitors or anti-angiogenics like sorafenib to improve efficacy. Furthermore, understanding the interaction between SSTR2 signaling and the tumor microenvironment—including stromal remodeling, immune evasion, and angiogenesis—could reveal combinatorial vulnerabilities that enhance treatment outcomes.

In summary, our findings establish a strong rationale for positioning SSTR2 as both a theranostic biomarker and a molecular target in HCC. Future studies should focus on validating these observations in large, independent cohorts and in vivo models, with an emphasis on preclinical investigations that evaluate SSTR2-targeted imaging and therapy in liver cancer. Ultimately, leveraging SSTR2 biology may open the door to personalized, receptor-guided treatment strategies that improve survival and quality of life for patients with advanced HCC.

## Data Availability

Data from this study are available upon reasonable request from the corresponding authors.

## Supplementary Materials

The following supporting information can be downloaded at: https://www.mdpi.com/article/10.3390/curroncol32090512/s1, Figure S1: Higher expression of SSTR2 in human malignancies compared to normal tissues; Figure S2: Higher expression of SSTR2 predicts a poor overall survival in HCC patients.

## Abbreviations

The following abbreviations are used in this manuscript:

HCC	Hepatocellular carcinoma
SSTR2	Somatostatin receptor 2

## References

[1] Brown Z.J., Tsilimigras D.I., Ruff S.M., Mohseni A., Kamel I.R., Cloyd J.M., Pawlik T.M. (2023). Management of Hepatocellular Carcinoma: A Review. JAMA Surg..

[2] Craig A.J., von Felden J., Garcia-Lezana T., Sarcognato S., Villanueva A. (2020). Tumour evolution in hepatocellular carcinoma. Nat. Rev. Gastroenterol. Hepatol..

[3] Siegel R.L., Miller K.D., Wagle N.S., Jemal A. (2023). Cancer statistics, 2023. CA Cancer J. Clin..

[4] Finn R.S., Qin S., Ikeda M., Galle P.R., Ducreux M., Kim T.Y., Kudo M., Breder V., Merle P., Kaseb A.O. (2020). Atezolizumab plus Bevacizumab in Unresectable Hepatocellular Carcinoma. N. Engl. J. Med..

[5] Safri F., Nguyen R., Zerehpooshnesfchi S., George J., Qiao L. (2024). Heterogeneity of hepatocellular carcinoma: From mechanisms to clinical implications. Cancer Gene Ther..

[6] Marin J.J.G., Macias R.I.R., Monte M.J., Romero M.R., Asensio M., Sanchez-Martin A., Cives-Losada C., Temprano A.G., Espinosa-Escudero R., Reviejo M. (2020). Molecular Bases of Drug Resistance in Hepatocellular Carcinoma. Cancers.

[7] Llovet J.M., Castet F., Heikenwalder M., Maini M.K., Mazzaferro V., Pinato D.J., Pikarsky E., Zhu A.X., Finn R.S. (2022). Immunotherapies for hepatocellular carcinoma. Nat. Rev. Clin. Oncol..

[8] Guenter R., Aweda T., Carmona Matos D.M., Jang S., Whitt J., Cheng Y.Q., Liu X.M., Chen H., Lapi S.E., Jaskula-Sztul R. (2020). Overexpression of somatostatin receptor type 2 in neuroendocrine tumors for improved Ga68-DOTATATE imaging and treatment. Surgery.

[9] Lehman J.M., Hoeksema M.D., Staub J., Qian J., Harris B., Callison J.C., Miao J., Shi C., Eisenberg R., Chen H. (2019). Somatostatin receptor 2 signaling promotes growth and tumor survival in small-cell lung cancer. Int. J. Cancer.

[10] He J.H., Wang J., Yang Y.Z., Chen Q.X., Liu L.L., Sun L., Hu W.M., Zeng J. (2021). SSTR2 is a prognostic factor and a promising therapeutic target in glioma. Am. J. Transl. Res..

[11] Lequoy M., Desbois-Mouthon C., Wendum D., Gupta V., Blachon J.L., Scatton O., Dumont S., Bonnemaire M., Schmidlin F., Rosmorduc O. (2017). Somatostatin receptors in resected hepatocellular carcinoma: Status and correlation with markers of poor prognosis. Histopathology.

[12] Kim J.Y., Kim J., Kim Y.I., Yang D.H., Yoo C., Park I.J., Ryoo B.Y., Ryu J.S., Hong S.M. (2024). Somatostatin receptor 2 (SSTR2) expression is associated with better clinical outcome and prognosis in rectal neuroendocrine tumors. Sci. Rep..

[13] Xu Y., Quan Z., Zhan Y., Wang H., Luo J., Wang W., Fan S. (2024). SSTR2 positively associates with EGFR and predicts poor prognosis in nasopharyngeal carcinoma. J. Clin. Pathol..

[14] Cerami E., Gao J., Dogrusoz U., Gross B.E., Sumer S.O., Aksoy B.A., Jacobsen A., Byrne C.J., Heuer M.L., Larsson E. (2012). The cBio cancer genomics portal: An open platform for exploring multidimensional cancer genomics data. Cancer Discov..

[15] Tang D., Chen M., Huang X., Zhang G., Zeng L., Zhang G., Wu S., Wang Y. (2023). SRplot: A free online platform for data visualization and graphing. PLoS ONE.

[16] Lanczky A., Gyorffy B. (2021). Web-Based Survival Analysis Tool Tailored for Medical Research (KMplot): Development and Implementation. J. Med. Internet Res..

[17] Llovet J.M., Pinyol R., Kelley R.K., El-Khoueiry A., Reeves H.L., Wang X.W., Gores G.J., Villanueva A. (2022). Molecular pathogenesis and systemic therapies for hepatocellular carcinoma. Nat. Cancer.

[18] Wang Y., Deng B. (2023). Hepatocellular carcinoma: Molecular mechanism, targeted therapy, and biomarkers. Cancer Metastasis Rev..

[19] Zheng J., Wang S., Xia L., Sun Z., Chan K.M., Bernards R., Qin W., Chen J., Xia Q., Jin H. (2025). Hepatocellular carcinoma: Signaling pathways and therapeutic advances. Signal Transduct. Target. Ther..

[20] Luo X., He X., Zhang X., Zhao X., Zhang Y., Shi Y., Hua S. (2024). Hepatocellular carcinoma: Signaling pathways, targeted therapy, and immunotherapy. MedComm.

[21] Dimri M., Satyanarayana A. (2020). Molecular Signaling Pathways and Therapeutic Targets in Hepatocellular Carcinoma. Cancers.

[22] Zhang H., Dai S.D., Zhang D., Liu D., Zhang F.Y., Zheng T.Y., Cui M.M., Dai C.L. (2014). Delta-catenin promotes the proliferation and invasion of colorectal cancer cells by binding to E-cadherin in a competitive manner with p120 catenin. Target. Oncol..

[23] Brabletz T., Kalluri R., Nieto M.A., Weinberg R.A. (2018). EMT in cancer. Nat. Rev. Cancer.

[24] Zheng Q., Tang Z.Y., Xue Q., Shi D.R., Song H.Y., Tang H.B. (2000). Invasion and metastasis of hepatocellular carcinoma in relation to urokinase-type plasminogen activator, its receptor and inhibitor. J. Cancer Res. Clin. Oncol..

[25] Ye L., Zhao X., Lu J., Qian G., Zheng J.C., Ge S. (2013). Knockdown of TIGAR by RNA interference induces apoptosis and autophagy in HepG2 hepatocellular carcinoma cells. Biochem. Biophys. Res. Commun..

[26] Hong J., Yuan Y., Wang J., Liao Y., Zou R., Zhu C., Li B., Liang Y., Huang P., Wang Z. (2014). Expression of variant isoforms of the tyrosine kinase SYK determines the prognosis of hepatocellular carcinoma. Cancer Res..

[27] Zhang L., Zhang C., Xing Z., Lou C., Fang J., Wang Z., Li M., He H., Bai H. (2022). Fibronectin 1 derived from tumor-associated macrophages and fibroblasts promotes metastasis through the JUN pathway in hepatocellular carcinoma. Int. Immunopharmacol..

[28] Lv S., Ning H., Li Y., Wang J., Jia Q., Wen H. (2020). Inhibition of cyclinB1 Suppressed the Proliferation, Invasion, and Epithelial Mesenchymal Transition of Hepatocellular Carcinoma Cells and Enhanced the Sensitivity to TRAIL-Induced Apoptosis. Onco Targets Ther..

[29] Zhao P.W., Zhang J.W., Liu Y., Liu Y., Liu J.W., Huang J.Z. (2020). SRC-1 and Twist1 are prognostic indicators of liver cancer and are associated with cell viability, invasion, migration and epithelial-mesenchymal transformation of hepatocellular carcinoma cells. Transl. Cancer Res..

[30] Moon H., Ro S.W. (2021). MAPK/ERK Signaling Pathway in Hepatocellular Carcinoma. Cancers.

[31] Che L., Paliogiannis P., Cigliano A., Pilo M.G., Chen X., Calvisi D.F. (2022). Corrigendum: Pathogenetic, Prognostic, and Therapeutic Role of Fatty Acid Synthase in Human Hepatocellular Carcinoma. Front. Oncol..

[32] Na T.Y., Schecterson L., Mendonsa A.M., Gumbiner B.M. (2020). The functional activity of E-cadherin controls tumor cell metastasis at multiple steps. Proc. Natl. Acad. Sci. USA.

[33] Tanaka S., Shiraha H., Nakanishi Y., Nishina S., Matsubara M., Horiguchi S., Takaoka N., Iwamuro M., Kataoka J., Kuwaki K. (2012). Runt-related transcription factor 3 reverses epithelial-mesenchymal transition in hepatocellular carcinoma. Int. J. Cancer.

[34] Krajnovic M., Kozik B., Bozovic A., Jovanovic-Cupic S. (2023). Multiple Roles of the RUNX Gene Family in Hepatocellular Carcinoma and Their Potential Clinical Implications. Cells.

[35] Morse M.A., Sun W., Kim R., He A.R., Abada P.B., Mynderse M., Finn R.S. (2019). The Role of Angiogenesis in Hepatocellular Carcinoma. Clin. Cancer Res..

[36] Stock P., Monga D., Tan X., Micsenyi A., Loizos N., Monga S.P. (2007). Platelet-derived growth factor receptor-alpha: A novel therapeutic target in human hepatocellular cancer. Mol. Cancer Ther..

[37] Zhang T., Sun H.C., Xu Y., Zhang K.Z., Wang L., Qin L.X., Wu W.Z., Liu Y.K., Ye S.L., Tang Z.Y. (2005). Overexpression of platelet-derived growth factor receptor alpha in endothelial cells of hepatocellular carcinoma associated with high metastatic potential. Clin. Cancer Res..

[38] Wilhelm S.M., Adnane L., Newell P., Villanueva A., Llovet J.M., Lynch M. (2008). Preclinical overview of sorafenib, a multikinase inhibitor that targets both Raf and VEGF and PDGF receptor tyrosine kinase signaling. Mol. Cancer Ther..

[39] Cheng A.L., Kang Y.K., Chen Z., Tsao C.J., Qin S., Kim J.S., Luo R., Feng J., Ye S., Yang T.S. (2009). Efficacy and safety of sorafenib in patients in the Asia-Pacific region with advanced hepatocellular carcinoma: A phase III randomised, double-blind, placebo-controlled trial. Lancet Oncol..

[40] Llovet J.M., Ricci S., Mazzaferro V., Hilgard P., Gane E., Blanc J.F., de Oliveira A.C., Santoro A., Raoul J.L., Forner A. (2008). Sorafenib in advanced hepatocellular carcinoma. N. Engl. J. Med..

[41] Li Y., Xia J., Shao F., Zhou Y., Yu J., Wu H., Du J., Ren X. (2021). Sorafenib induces mitochondrial dysfunction and exhibits synergistic effect with cysteine depletion by promoting HCC cells ferroptosis. Biochem. Biophys. Res. Commun..

[42] Liu L., Cao Y., Chen C., Zhang X., McNabola A., Wilkie D., Wilhelm S., Lynch M., Carter C. (2006). Sorafenib blocks the RAF/MEK/ERK pathway, inhibits tumor angiogenesis, and induces tumor cell apoptosis in hepatocellular carcinoma model PLC/PRF/5. Cancer Res..

[43] Chen S.W., Lin L.C., Kuo Y.C., Liang J.A., Kuo C.C., Chiou J.F. (2014). Phase 2 study of combined sorafenib and radiation therapy in patients with advanced hepatocellular carcinoma. Int. J. Radiat. Oncol. Biol. Phys..

[44] Momeny M., AghaAmiri S., Hernandez Vargas S., Acidi B., Ghosh S.C., Bateman T.M., Adams J.T., Khalaj V., Kaseb A.O., Tran Cao H.S. (2025). SSTR2-targeted theranostics in hepatocellular carcinoma. Cancers.

[45] Hashiguchi M., Ueno S., Sakoda M., Iino S., Hiwatashi K., Minami K., Ando K., Mataki Y., Maemura K., Shinchi H. (2013). Clinical implication of ZEB-1 and E-cadherin expression in hepatocellular carcinoma (HCC). BMC Cancer.

[46] Zhao S., Li H., Wang Q., Su C., Wang G., Song H., Zhao L., Luan Z., Su R. (2015). The role of c-Src in the invasion and metastasis of hepatocellular carcinoma cells induced by association of cell surface GRP78 with activated alpha2M. BMC Cancer.

[47] Mo S., Fang D., Zhao S., Thai Hoa P.T., Zhou C., Liang T., He Y., Yu T., Chen Y., Qin W. (2022). Down regulated oncogene KIF2C inhibits growth, invasion, and metastasis of hepatocellular carcinoma through the Ras/MAPK signaling pathway and epithelial-to-mesenchymal transition. Ann. Transl. Med..

[48] Llovet J.M., Kelley R.K., Villanueva A., Singal A.G., Pikarsky E., Roayaie S., Lencioni R., Koike K., Zucman-Rossi J., Finn R.S. (2021). Hepatocellular carcinoma. Nat. Rev. Dis. Primers.

[49] Deppen S.A., Liu E., Blume J.D., Clanton J., Shi C., Jones-Jackson L.B., Lakhani V., Baum R.P., Berlin J., Smith G.T. (2016). Safety and Efficacy of 68Ga-DOTATATE PET/CT for Diagnosis, Staging, and Treatment Management of Neuroendocrine Tumors. J. Nucl. Med..

[50] Poeppel T.D., Binse I., Petersenn S., Lahner H., Schott M., Antoch G., Brandau W., Bockisch A., Boy C. (2011). 68Ga-DOTATOC versus 68Ga-DOTATATE PET/CT in functional imaging of neuroendocrine tumors. J. Nucl. Med..

[51] Delbart W., Karabet J., Marin G., Penninckx S., Derrien J., Ghanem G.E., Flamen P., Wimana Z. (2022). Understanding the Radiobiological Mechanisms Induced by (177)Lu-DOTATATE in Comparison to External Beam Radiation Therapy. Int. J. Mol. Sci..

[52] Strosberg J., El-Haddad G., Wolin E., Hendifar A., Yao J., Chasen B., Mittra E., Kunz P.L., Kulke M.H., Jacene H. (2017). Phase 3 Trial of (177)Lu-Dotatate for Midgut Neuroendocrine Tumors. N. Engl. J. Med..

