# Supplementary figures and images for: Somatostatin Receptor 2 Overexpression in Hepatocellular Carcinoma: Implications for Cancer Biology and Therapeutic Applications
Source: Curr Oncol. 2025 Sep 13;32(9):512. doi: 10.3390/curroncol32090512 (PMC12468542; doi:10.3390/curroncol32090512)

SSTR2 gene expression

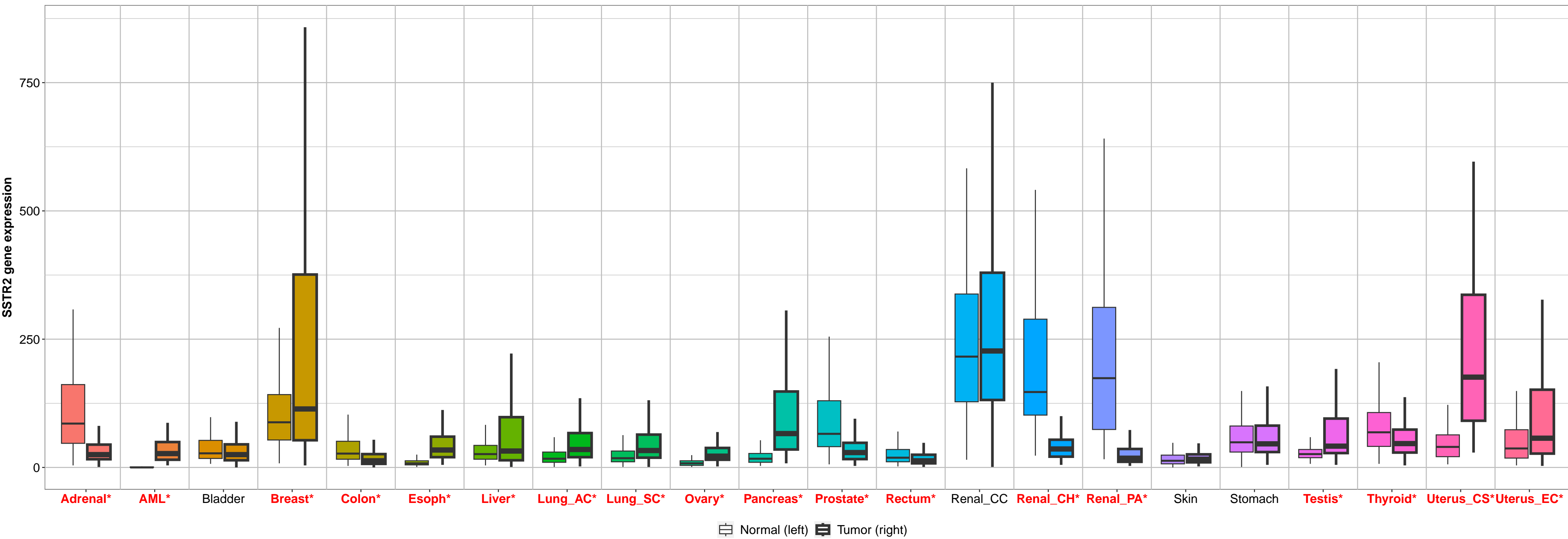

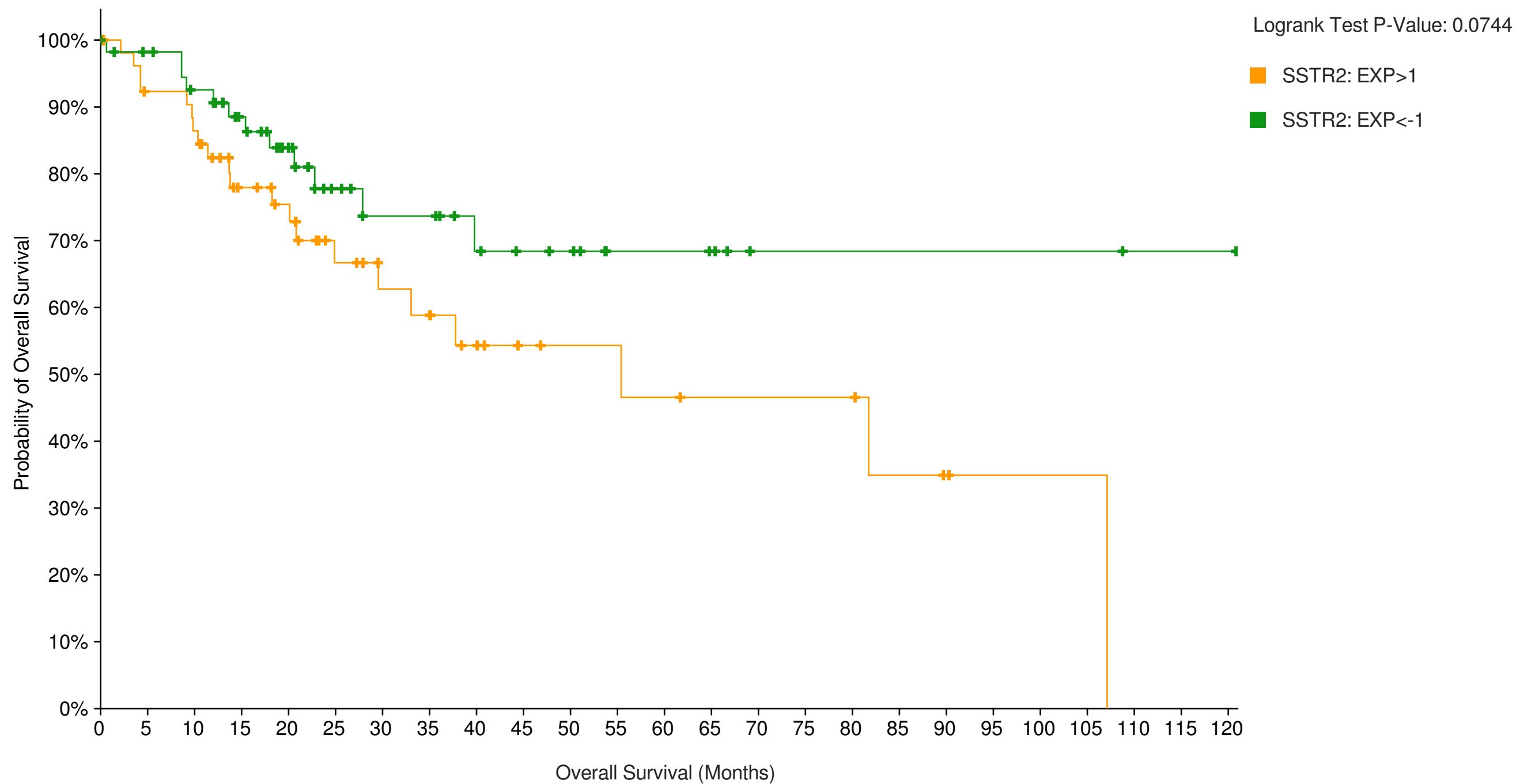[illegible]

Supplement: Supplementary file 1 [file curroncol-32-00512-s001.zip › curroncol-3832138-supplementary.pdf]
